# Smart Aging Platform for Evaluating Cognitive Functions in Aging: A Comparison with the MoCA in a Normal Population

**DOI:** 10.3389/fnagi.2017.00379

**Published:** 2017-11-21

**Authors:** Sara Bottiroli, Cristina Tassorelli, Marialisa Lamonica, Chiara Zucchella, Elena Cavallini, Sara Bernini, Elena Sinforiani, Stefania Pazzi, Paolo Cristiani, Tomaso Vecchi, Daniela Tost, Giorgio Sandrini

**Affiliations:** ^1^Headache Science Centre, C. Mondino National Neurological Institute, Pavia, Italy; ^2^Department of Brain and Behavioral Sciences, University of Pavia, Pavia, Italy; ^3^Associazione Smart Aging, Catanzaro, Italy; ^4^UOC Neurologia A Azienda Ospedaliera Universitaria Integrata, Verona, Italy; ^5^Consorzio di Bioingegneria e Informatica Medica, Pavia, Italy; ^6^Computer Graphics Division CREB, Universitat Politecnica de Catalunya, Barcelona, Spain

**Keywords:** virtual reality, serious games, cognitive assessment, global cognitive functions, normal aging

## Abstract

**Background:** Smart Aging is a Serious games (SGs) platform in a 3D virtual environment in which users perform a set of screening tests that address various cognitive skills. The tests are structured as 5 tasks of activities of daily life in a familiar environment. The main goal of the present study is to compare a cognitive evaluation made with Smart Aging with those of a classic standardized screening test, the Montreal Cognitive Assessment (MoCA).

**Methods:** One thousand one-hundred thirty-one healthy adults aged between 50 and 80 (*M* = 64.3 ± 8.3) were enrolled in the study. They received a cognitive evaluation with the MoCA and the Smart Aging platform. Participants were grouped according to their MoCA global and specific cognitive domain (i.e., memory, executive functions, working memory, visual spatial elaboration, language, and orientation) scores and we explored differences among these groups in the Smart Aging indices.

**Results:** One thousand eighty-six older adults (*M* = 64.0 ± 8.0) successfully completed the study and were stratified according to their MoCA score: Group 1 with MoCA < 27 (*n* = 360); Group 2 with 27 ≥ MoCA < 29 (*n* = 453); and Group 3 with MoCA ≥ 29 (*n* = 273). MoCA groups significantly differed in most of the Smart Aging indices considered, in particular as concerns accuracy (*p*s < 0.001) and time (*p*s < 0.001) for completing most of the platform tasks. Group 1 was outperformed by the other two Groups and was slower than them in these tasks, which were those supposed to assess memory and executive functions. In addition, significant differences across groups also emerged when considering the single cognitive domains of the MoCA and the corresponding performances in each Smart Aging task. In particular, this platform seems to be a good proxy for assessing memory, executive functions, working memory, and visual spatial processes.

**Conclusion:** These findings demonstrate the validity of Smart Aging for assessing cognitive functions in normal aging. Future studies will validate this platform also in the clinical aging populations.

## Introduction

Aging is associated with an increased risk of cognitive impairment, for which early diagnosis becomes a critical aspect. This consideration, together with the substantial increase in the proportion of older adults in the general population, has determined a growing need and interest in screening tools able to detect cognitive changes that can be easily administered and distributed. To date, common cognitive evaluations are based on paper and pencil neuropsychological evaluations, which require high logistical and personnel-related costs (Kang et al., 2008). In addition, neuropsychological tests are characterized by the limit of having a moderate level of ecological validity when predicting real-word cognitive performance, together with the fact of being too psychologically stressful with the risk of producing skewed results (Chaytor and Schmitter-Edgecombe, 2003). For all these reasons, these tests do not embrace the advantages that can be excellently provided by new technologies that are dramatically advancing in the last years.

Serious games (SGs) based on Virtual Reality (VR) are innovative computer games designed for purposes other than pure leisure (Charsky, 2010). They usually consist in the presentation of 3D realistic scenarios where real-life situations can be simulated, allowing to enhance ecological validity with respect to traditional cognitive assessments. SGs have been used in many contexts (i.e., education, training, and simulation) and, recently, they have been proposed also in the health domain for the assessment and rehabilitation of psychiatric and neuropsychological conditions. In particular, in the field of neuropsychological assessment, SGs have numerous features that make them an interesting tool for the detection of cognitive impairments and for overcoming the limitations of other traditional types of measures. They, indeed, have the potential to stimuli more discretely and with greater precision than the traditional tests (Tong et al., 2016). Furthermore, the SGs approach allows the evaluation of multiple cognitive aspects of the subjects' responses, i.e., memory and executive functions. SGs can also be self-administered or require little training, provide a pleasant experience and reduce the psychological stress caused by the traditional screening tools (Ismail et al., 2010). All these aspects are particularly relevant for the diagnosis of the first phases of cognitive impairments. Therefore, SGs can be used to perform large scale, low-cost screening campaigns of cognitive functions of the yielding to earlier detection of cognitive impairments and anticipated enrolment in rehabilitation programs.

Most of studies in this field of research have evaluated the effectiveness of SGs for the rehabilitation of cognitive functions in normal and pathological aging (Gamberini et al., 2006; Anguera et al., 2013; and see Wiemeyer and Kliem, 2012; Robert et al., 2014 for a review), whereas still little is known about their functioning in the assessment of the cognitive status in these populations. The studies published so far (see Valladores-Rodríguez et al., 2016 for a review) differ greatly in the approach adopted, with some of them creating computerized versions of traditional cognitive tests (see for a review Wild et al., 2008; Zygouris and Tsolaki, 2014) or adapting existing leisure games for assessing cognition (Aalbers et al., 2013; Tong et al., 2016). In this field, what is particularly interesting is the use of SGs for replicating real life situations, which deserves further exploration. Many of the existing studies focused on the early detection of cognitive impairment (e.g., Tarnanas et al., 2013, 2014, 2015; Manera et al., 2015; Zygouris et al., 2015; Tong et al., 2016; Fernandez-Montenegro and Argyriou, 2017; Vallejo et al., 2017), whereas little is known about the use of that platforms in normal aging as accurate screening measures of cognitive functions. To date, indeed, most of the studies were only at a preliminary basis, were tested on small samples of older adults and involved daily live activities that could be gender-oriented. For instance, McGee et al. (2000) developed a virtual environment version of the Morris Water Task—in which participants navigated in a virtual water pool—and tested its ability in assessing visuospatial mechanisms in a sample of 30 older adults aged between 65 and 92 years old. Recently Boletsis and McCallum (2016) verified the association between a SG called Smartkuber—consisting in a collection of cognitive mini-games in augmented reality—and standard cognitive assessments in a sample of 13 older adults. Another virtual environment platform for cognitive assessment was the ECO-VR (Oliveira et al., 2016), which consisted in performing several real-word activities, such as listening to a message on the answering machine, and that was tested for correlations with standard neuropsychological assessments in a series of pilot studies involving a sample of 37 older adults. Other examples in this field are more recently represented by Vallejo et al. (2017) that developed a SG platform for assessing cognitive processes and everyday life consisting in a virtual cooking scenario, and Davison et al. (2017) with a virtual parking simulator for cognitive evaluations. Taken together, all these studies are interesting as they show the promising applications of virtual realities and serious games for assessing cognition. However, it is evident that more research is necessary to investigate in greater detail their use for cognitive assessment in normal aging.

To this end, we have recently developed Smart Aging, a platform for the assessment of cognitive functions based on the SG technology (Pazzi et al., 2014; Tost et al., 2014, 2015). The various games consisted in 3D real life tasks developed to assess global cognition and specific cognitive mechanisms, such as episodic and prospective memory, attention, and executive functions, being those more impaired in dementia (Stopford et al., 2012). Smart Aging is the operationalized version of a prototype that has been adapted and revised following pilot testing in our research institute, within a sample of individuals with varying age, education level, and sex (Zucchella et al., 2014). The game was designed in order to be easily self-administered also to non-expert users just following the instructions that precede each task, and the navigation in 3D was made particularly accessible thanks to the use of a touch-screen.

With the ultimate intention of developing an assessment tool for the evaluation of cognition as a whole in an ecological context, in the present study we aimed at validating Smart Aging (Pazzi et al., 2014; Tost et al., 2014, 2015; Zucchella et al., 2014) on a sample of 1086 healthy older adults.

## Materials and methods

### Design of the comparative study

Many approaches can be adopted in order to validate a game, such as evaluate its sensitivity and specificity in detecting a particular disorder or by comparing performances here with those of traditional measures. In the present study, to evaluate the reliability of Smart Aging in assessing even the subtle differences of cognitive functions that can be found in a normal population, we stratified subjects in 3 subgroups defined by their MoCA scores (i.e., <27; 27≥ and <29; ≥29) and subsequently compared the performances in each specific Smart Aging task with MoCA scores (Nasreddine et al., 2005). We chose the MoCA as our reference screening tool as it proved to be particularly sensitive to detect early cognitive impairment and early dementia as well as it is an accurate screening measurement of cognitive ability. When compared to other measures of cognitive screening such as MMSE, MoCA resulted better in detecting age-related cognitive decline (Gluhm et al., 2013; Ciesielska et al., 2016). Furthermore, the MoCA allows the reliable evaluation of several cognitive domains, such as executive functions, memory, orientation, language, visuo-spatial abilities, and working memory.

### Participants

We set out to validate Smart Aging in a cohort of healthy 1,131 community-dwelling individuals recruited across Italy aged 50 years and older (age range 50–80, *M* = 64.3 ± 8.3). All participants were volunteers recruited from public entities, universities of the third age, social clubs, etc. They all lived independently, were reasonably fit and healthy, and had active social and cognitive lives. Italian was the mother tongue for all participants in the study. Inclusion criteria were age comprised between 50 and 80, a MoCA score ≥26 and absence of current psychiatric and neurological illness or substance abuse. Forty-five participants were excluded because they scored lower than 26 at the MoCA. Hence the final sample comprised 1,086 older adults (*M* = 64.0 ± 8.0). Written informed consent form was obtained and the study was approved by the local Ethics Committee. Participants' characteristics are reported in Table 1.

**Table 1:** Demographic characteristics of the population investigated in general and in the 3 subgroups identified on the basis of the MoCA score.

	**Total** **(*n* = 1,086)**	**MoCA < 27** **(*n* = 360)**	**27≥MoCA<29** **(*n* = 453)**	**MoCA ≥ 29** **(*n* = 273)**	***p***
Age	64.0 (8.3)	67.1 (7.9)	63.1 (8.1)	61.6 (7.7)	<0.001
Gender					0.56
F	608 (56.0%)	206 (57.2%)	245 (54.1%)	157 (57.5%)	
M	478 (44.0%)	154 (42.8%)	208 (45.9%)	116 (42.5%)	
Education					<0.001
Primary school	266 (24.5%)	145 (40.3%)	88 (19.4%)	33 (12.1%)	
Middle school	299 (27.5%)	116 (32.2%)	123 (27.2%)	60 (22.0%)	
High school	289 (26.6%)	62 (17.2%)	131 (28.9%)	96 (35.2%)	
University	232 (21.4%)	37 (10.3%)	111 (24.5%)	84 (30.8%)	
MoCA score	27.8 (1.4)	26.3 (0.5)	28.0 (0.7)	29.6 (0.5)	<0.001

### Neuropsychological assessment

Before receiving the Smart Aging test, participants underwent the Montreal Cognitive Assessment (Nasreddine et al., 2005) that was administered by a trained neuropsychologist. The MoCA is a 30-point test comprising a series of cognitive subtests, pertaining to six different cognitive domains. First, short-term memory recall task involves two learning trials of five nouns and delayed recall after 5 min. Second, visuospatial abilities are assessed using a clock-drawing task and a three-dimensional cube copy. Third, executive functions are assessed using an alternation task taken from the Trail Making B task, a phonemic fluency task, and a two-item verbal abstraction task. Fourth, attention, concentration, and working memory are evaluated using a sustained attention task (target detection using tapping), a serial subtraction task, and digits forward and backward. Fifth, language is assessed using a three-item confrontation naming task with low-familiarity animals, repetition of two syntactically complex sentences, and the aforementioned fluency task. Sixth, orientation to time and place is also evaluated.

### The smart aging platform

Smart Aging is a SG platform developed from the collaboration of a multidisciplinary team comprising neurologists, psychologists, neuropsychologists, bioinformatics, designers, and ICT engineers. Detailed technical information of this platform is reported elsewhere (Pazzi et al., 2014; Tost et al., 2014, 2015; Zucchella et al., 2014). Older adults are sitting in front of a desktop personal computer equipped with a sound card, where they navigate through and interact with the environment by using a touch screen monitor. The application is based on a first-person paradigm so there is no user 3D avatar. The virtual position of the user within the environment is associated with a camera and the navigation model allows users to move within the environment at a constant height over the floor plane and to rotate the camera (head) within a limited range of angles.

**Figure 1:**
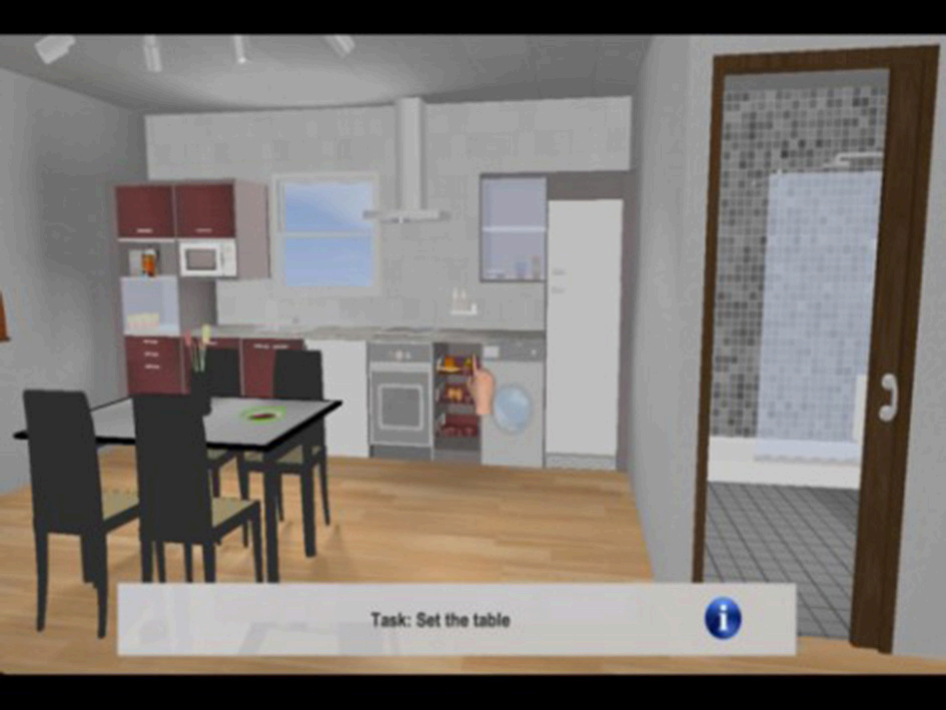
An example of the virtual scenario used in the Smart Aging platform.

The virtual 3D environment consists in a loft assembling in a reduced space the basic elements of interaction of a private home environment: a kitchen corner, a room corner and a living room corner (Figure 1).

The virtual environment is equipped with the following elements:

- Fixed elements that do not allow any interaction: walls, floor, ceiling, windows, and decorative elements such as paintings, curtains, and carpets;
- Fixed elements that cannot be moved but can be used as a top to put and pick movable objects: bed, table, coach, kitchen marble, shelves;
- Container elements with doors that can be opened and closed to put or get: kitchen cupboards, fridge, and wardrobe;
- Special interactive elements with specific functionalities: burners, sink;
- Movable elements such as clothes, books, and food.

The environment is framed by a layer able to show 2D information such as instructions, scoring feedback and miniatures. The 2D layer also allow 2D user-interaction.

### Smart aging tasks

Smart Aging platform have been designed to engage in task-specific scenarios where participants are asked to perform 5 tasks closely related to daily life activities. The tasks have been developed in order to evaluate different cognitive functions: executive functions (reasoning and planning), attention (selected and divided), memory (short and long term, prospective), and visuo-spatial orientation. A description of tasks with the indication of the cognitive functions involved is reported in Table 2.

**Table 2:** The Smart Aging tasks.

**Task**	**Cognitive functions**
**Task 1—Objects search**	
After an exploration of the kitchen, the subject is asked to look for a list of objects	Memory, spatial orientation and attention
**Task 2—Water the flowers while listening to the radio**	
The subject is asked to turn on the radio and press the spacebar every time the word “sun” is aired, while watering the flowers on the windowsill in the dining room	Executive functions (planning), divided attention (dual task)
**Task 3—Make a phone call**	
The person is asked to make a phone call using the phone book and the phone placed on the night table next to the bed. The subject is asked to remember to turn the TV on, once the number is dialled	Executive functions, selective attention, working memory, prospective memory
**Task 4—Choose the right object**	
A 2D screen with 24 images of objects is shown. The subjects has to identify the 12 objects presented in Task 1	Memory (recognition)
**Task 5—Find the objects**	
The subject is positioned in front of the kitchen, and he/she is asked to find each of the objects that he looked for in Task 1	Long-term memory (recall), spatial orientation and attention

Each task is preceded by written instructions on the screen explaining to the subject what he/she is requested to do; the instruction is visible on the screen for up to 30 s and the subjects can start the task when ready. Before the evaluative session proper, subjects naïve for the use of ICT and touch screen, assist to a 10-min interactive demo to familiarize with the virtual environment and the functioning of the touch screen. No other feedback is provided while the subjects are performing the games. The execution of all the games, including the familiarization demo, requires from 10 to 30 min. The system records various measures (positions, times, and actions) while the participant experiences the virtual environment and performs the tasks. Scores provide a picture of participants' cognitive functions.

In particular, the game records all users' actions and computes a set of indices for each task separately. For each of the five tasks we always considered accuracy, time, and distance. As for accuracy, for Tasks 1, 4, and 5, it referred to the total number of objects correctly remembered, whereas for Tasks 2 and 3 to the total number of correct actions while completing each of these tasks. For Task 3, we also considered the correct recall of the telephone number necessary for making the phone call, as well as the action of prospective memory, that is, the fact that each participant remembered to set on the TV at the end of the task. As for time, this index referred to the time needed to accomplish each task, from its start to the end. As for distance, it refers to the amount of meters covered in the loft while performing each task, from its start to the end. The only exception was represented by Task 2, in which we had two separated indices of distance: (1) without interference—i.e., the distance covered while listening the radio only—(2) with interference—i.e., the distance covered while listening the radio and watering flowers.

### Statistical analysis

For each Smart Aging task, we considered accuracy, reaction time and distance, which were converted in *z*-score units. We also computed a total score for each task, given from the sum (or difference for reverted scores, such as reaction time and distance) of the scores for that task. Furthermore, we computed a total score, obtained from the total scores of all five tasks, named Smart Aging total score.

Participants were classified into three groups according to their MoCA scores: Group 1 with MoCA < 27 (*n* = 360); Group 2 with 27≥MoCA < 29 (*n* = 453); and Group 3 with MoCA≥29 (*n* = 273). A further classification was made according to participants' performance in each of the six MoCA cognitive domains. Hence, for each of these domains, we further grouped participants in Subgroup 1, which was composed by subjects scoring under the median for that domain, and Subgroup 2, with those scoring equal or over the median. We used univariate analysis of variance (ANOVA) in order to compare groups normally distributed variables. As the distribution of the data for Smart Aging was not normal, group comparisons were performed using non-parametric Kruskal–Wallis tests followed by Mann-Whitney *U*-tests corrected for multiple comparisons. In order to verify whether scores in the game were in line with the standard neuropsychological assessment, we submitted them to separate Mann-Whitney *U*-tests, with MoCA groups as independent between-subject factors. A series of receiver operating characteristics (ROC) analyses were performed to evaluate the relationship between sensitivity and specificity of the total scores in the five tasks and in the Smart Aging total score for the identification of MoCA subgroups. To this end, we considered Group 1 (the group with the lowest MoCA score) vs. Group 2 + Group 3, and Group 3 (the group with the highest MoCA score) vs. Group 1 + Group 2. The area under the ROC curve (AUC) gives the proportion of cases that are correctly discriminated by the considered variables. A further series of Mann-Whitney *U*-tests was carried out on each Smart Aging score considering classifications made according MoCA subtests. Effects size were calculated by using G^*^Power 3 (Faul et al., 2007). Pearson's Chi-square was used for categorical variables. An alpha of 0.05 was used for all parametric tests, while a significance level of 0.017 (0.05/3) was applied for non-parametric tests involving three groups. The SPSS 23.0 statistical software package was used to perform all the statistical analysis.

## Results

### Participants characteristics

Demographic characteristics of participants are presented in Table 1. All three MoCA groups were significantly different in terms of age, *F*_(2, 1, 083)_ = 41.67, *p* < 0.001, being the Group 1 the oldest and Group 3 the youngest. The proportion of female and male participants was similar across groups, χ2 = 1.145; *d.f*. = 2; *p* = 0.56. Groups were instead differently balanced in terms of education, χ2 = 118.92; *d.f*. = 6; *p* < 0.001.

### Differences in smart aging scores according to MoCA groups

Means and standard deviations for the five Smart Aging tasks as a function of MoCA groups are reported in Table 1.

#### Task 1—object search

The 3 MoCA groups differed among them in all indices concerning Task 1 (see Table 3). As regards accuracy [χ^2^_(2)_ = 14.07, *p* = 0.001], Group 1 was outperformed by both Group 2 (*U* = 73777, *p* = 0.012, *d* = 0.20) and Group 3 (*U* = 41446, *p* < 0.001, *d* = 0.39), which did not differ between them (*U* = 58062, *p* = 0.13). As regards timing [χ^2^_(2)_ = 18.32, *p* < 0.001], Group 1 was slower than both Group 2 (*U* = 78038, *p* = 0.01, *d* = 0.18) and Group 3 (*U* = 39758, *p* < 0.001, *d* = 0.35), which were also different between them because Group 2 was slower than Group 3 (*U* = 55509, *p* = 0.02, *d* = 0.18). For distance [χ^2^_(2)_ = 10.42, *p* = 0.005], Group 1 also covered more distance than both Group 2 (*U* = 72141, *p* = 0.005, *d* = 0.15) and Group 3 (*U* = 42871, *p* = 0.006, *d* = 0.18), which did not differ between them (*U* = 61264, *p* = 0.83).

**Table 3:** Z Scores in the five Smart Aging tasks according to MoCA groups.

	**Total** **(*n* = 1,086)**	**Group 1** **(*n* = 370)**	**Group 2** **(*n* = 453)**	**Group 3** **(*n* = 273)**	***p***
**Task 1**					
Accuracy	0.05 (0.95)	−0.13^*^^+^ (1.10)	0.07^*^ (0.93)	0.23^+^ (0.68)	0.001
Time	−0.03 (0.98)	0.13^*^^+^ (1.08)	−0.05^*^^z^ (0.95)	−0.21^+^^z^ (0.85)	<0.001
Distance	0.01 (0.99)	0.13^*^^+^ (1.03)	−0.02^*^ (0.99)	−0.05^+^ (0.92)	0.005
Total	0.06 (1.93)	−0.40^*^^+^ (2.18)	0.17^*^^z^ (1.85)	0.51^+^^z^ (1.55)	<0.001
**Task 2**					
Accuracy	−0.01 (0.97)	−0.22^*^^+^ (1.07)	0.12^*^^z^ (0.94)	0.02^+^^z^ (0.86)	<0.001
Time	−0.03 (0.98)	0.05 (1.11)	−0.01 (0.94)	−0.18 (0.83)	0.08
Distance (without interference)	−0.00 (1.00)	−0.00 (0.92)	0.02 (1.16)	−0.04 (0.80)	0.41
Distance (with interference)	−0.00 (0.99)	0.02 (1.18)	0.01 (0.95)	−0.08 (0.73)	0.67
Total	0.02 (1.95)	−0.31^*^^+^ (2.20)	0.10^*^ (1.91)	0.34^+^ (1.57)	<0.001
**Task 3**					
Accuracy	0.01 (0.99)	−0.15^*^^+^ (1.08)	−0.06^*^ (0.93)	0.15^+^ (0.92)	<0.001
Accuracy of telephone number	0.00 (0.99)	−0.17^*^^+^ (1.10)	0.05^*^ (0.95)	0.13^+^ (0.88)	<0.001
Action of prospective memory	0.01 (0.99)	−0.16^*^^+^ (1.07)	0.06^*^ (0.96)	0.16^+^ (0.88)	<0.001
Time	−0.03 (0.99)	0.11^+^ (1.08)	−0.04^z^ (0.92)	−0.18^+^^z^ (0.94)	0.001
Distance	0.02 (0.94)	0.03 (1.02)	0.02 (0.84)	0.00 (1.00)	0.34
Total	0.04 (3.34)	−0.63^*^^+^ (3.72)	0.21^*^^z^ (3.14)	0.64^+^^z^ (2.99)	<0.001
**Task 4**					
Accuracy	0.01 (0.99)	−0.20^*^^+^ (1.01)	0.06^*^^z^ (1.01)	0.19^+^^z^ (0.90)	<0.001
Time	−0.01 (1.00)	0.11^+^ (1.03)	−0.00^z^ (1.00)	−0.18^+^^z^ (0.94)	0.001
Total	0.02 (1.58)	−0.31^*^^+^ (1.59)	0.06^*^^z^ (1.59)	0.39^+^^z^ (1.46)	<0.001
**Task 5**					
Accuracy	0.08 (0.78)	0.02 (0.96)	0.13 (0.62)	0.07 (0.75)	0.15
Time	−0.06 (0.91)	0.01 (0.98)	−0.09 (0.87)	−0.14 (0.88)	0.13
Distance	−0.05 (0.94)	0.00 (1.00)	−0.07 (0.93)	−0.08 (0.88)	0.57
Total	0.02 (2.04)	0.01 (2.29)	0.30 (1.83)	0.30 (1.99)	0.86
Smart Aging Total	0.34 (6.82)	−1.65^*^^+^ (7.91)	0.84^*^^z^ (6.14)	2.17^+^^z^ (5.59)	<0.001

*Table reports means and (standard deviations). Group 1: MoCA score < 27; Group 2: 27 ≤ MoCA < 29; Group 3: MoCA ≥ 29*.

* Denotes significant differences between Group 1 and Group 2;

+ Denotes significant differences between Group 1 and Group 3;

z *Denotes significant differences between Group 2 and Group 3*.

As regards Task 1 total score [χ^2^_(2)_ = 28.00, *p* < 0.001], Group 1 scored less than both Group 2 (*U* = 69913, *p* < 0.001, *d* = 0.28) and Group 3 (*U* = 37479, *p* < 0.001, *d* = 0.48), which were also different between them because Group 2 scored less than Group 3 (*U* = 55499, *p* = 0.021, *d* = 0.20).

First, the ROC curve and the AUC were measured for the total scores in the five tasks as well as for the Smart Aging total score by comparing Group 1 vs. Group 2 + Group 3. The AUC ranged from 0.572 to 0.611. Then, we measured the ROC curve by comparing Group 3 vs. Group 2 + Group 1, again the AUC ranged from 0.555 to 0.605. Both analyses indicated a poor individual discrimination capacity.

#### Task 2—water the flowers while listening to the radio

The 3 MoCA groups differed among them only for task accuracy [χ^2^_(2)_ = 24.34, *p* < 0.001], with Group 1 was outperformed by both Group 2 (*U* = 65527, *p* < 0.001, *d* = 0.34) and Group 3 (*U* = 43055, *p* = 0.007, *d* = 0.25), which also differed between them (*U* = 56806, *p* = 0.049, *d* = 0.11). As for timing [χ^2^_(2)_ = 1.77, *p* = 0.079] and distance without [χ^2^_(2)_ = 1.77, *p* = 0.41] or with interference [χ^2^_(2)_ = 0.81, *p* = 0.67], no significant differences resulted among groups.

As regards Task 2 total score [χ^2^_(2)_ = 16.35, *p* < 0.001], Group 1 scored less than both Group 2 (*U* = 71595, *p* < 0.001, *d* = 0.20) and Group 3 (*U* = 40386, *p* < 0.001, *d* = 0.34), which did not differ between them (*U* = 58407, *p* = 0.21).

#### Task 3—make a phone call

The 3 MoCA groups differed among them in all indices concerning Task 3, except for distance [χ^2^_(2)_ = 2.17, *p* = 0.34]. As for task accuracy [χ^2^_(2)_ = 18.16, *p* < 0.001], Group 1 was outperformed by both Group 2 (*U* = 73802, *p* = 0.003, *d* = 0.09) and Group 3 (*U* = 42220, *p* < 0.001, *d* = 0.30), which did not differ between them (*U* = 58899, *p* = 0.13). As for timing [χ^2^_(2)_ = 13.84, *p* = 0.001], Group 3 was faster than both Group 1 (*U* = 40894, *p* < 0.001, *d* = 0.29) and Group 2 (*U* = 55211, *p* = 0.016, *d* = 0.24), which were similar between them (*U* = 75713, *p* = 0.08). As for the telephone number to be called [χ^2^_(2)_ = 17.43, *p* < 0.001], Group 1 showed a better performance than both Group 2 (*U* = 73923, *p* = 0.001, *d* = 0.21) and Group 3 (*U* = 42963, *p* < 0.001, *d* = 0.30), which did not differ between them (*U* = 59838, *p* = 0.26). As for prospective memory [χ^2^_(2)_ = 18.84, *p* < 0.001], less participants in Group 1 remembered to perform the action of prospective memory than both Group 2 (*U* = 73626, *p* = 0.002, *d* = 0.22) and Group 3 (*U* = 42126, *p* < 0.001, *d* = 0.33), which did not differ between them (*U* = 59010, *p* = 0.14).

As regards Task 3 total score [χ^2^_(2)_ = 30.37, *p* < 0.001], Group 1 scored less than both Group 2 (*U* = 70164, *p* = 0.001, *d* = 0.24) and Group 3 (*U* = 36986, *p* < 0.001, *d* = 0.24), which were also different between them because Group 2 scored less than Group 3 (*U* = 54322, *p* = 0.006, *d* = 0.14).

#### Task 4—choose the right object

The 3 MoCA groups differed among them in both indices concerning Task 4. As for accuracy [χ^2^_(2)_ = 46.20, *p* < 0.001], Group 1 was outperformed by both Group 2 (*U* = 65978, *p* < 0.001, *d* = 026) and Group 3 (*U* = 35611, *p* < 0.001, *d* = 0.41), which were also different between them as Group 3 outperformed Group 2 (*U* = 56442, *p* < 0.001). As for timing [χ^2^_(2)_ = 15.06, *p* = 0.001], Group 3 was faster than both Group 1 (*U* = 40331, *p* < 0.001, *d* = 0.30) and Group 2 (*U* = 54945, *p* = 0.012, *d* = 0.19), which were also different between them (*U* = 75958, *p* = 0.09).

As for Task 4 total score [χ^2^_(2)_ = 28.12, *p* < 0.001], Group 1 scored less than both Group 2 (*U* = 69199, *p* < 0.001, *d* = 0.23) and Group 3 (*U* = 35257, *p* < 0.001, *d* = 0.46), which were also different between them because Group 2 scored less than Group 3 (*U* = 53524, *p* = 0.002, *d* = 0.22).

#### Task 5—find the objects

The three MoCA groups did not differ in any of the indices concerning Task 5: accuracy [χ^2^_(2)_ = 3.77, *p* = 0.15], timing [χ^2^_(2)_ = 4.09, *p* = 0.13], or distance covered [χ^2^_(2)_ = 1.14, *p* = 0.57].

As regards Task 5 total score [χ^2^_(2)_ = 3.09, *p* = 0.21], Groups did not differ among them.

#### Smart aging total score

As for this score [χ^2^_(2)_ = 55.99, *p* < 0.001], Group 1 scored less than both Group 2 (*U* = 63632, *p* < 0.001, *d* = 0.35) and Group 3 (*U* = 32902, *p* < 0.001, *d* = 0.56), which were also different between them because Group 2 scored less than Group 3 (*U* = 54615, *p* = 0.008, *d* = 0.23).

### Differences in smart aging scores according to MoCA memory-domain subgroups

Means and standard deviations for the five Smart Aging tasks as a function of MoCA memory-domain subgroups are reported in Table 4.

**Table 4:** Z Scores in the five Smart Aging tasks according to MoCA memory-domain subgroups.

	**Subgroup 1** **(*n* = 484)**	**Subgroup 2** **(*n* = 602)**	***p***
**Task 1**			
Accuracy	−0.06^*^ (1.05)	0.14^*^ (0.86)	0.004
Time	0.04^*^ (1.05)	−0.09^*^ (0.93)	0.03
Distance	0.09^*^ (1.01)	−0.04^*^ (0.98)	0.025
**Task 2**			
Accuracy	−0.03 (1.01)	−0.01 (0.95)	0.86
Time	0.02 (1.01)	−0.07 (0.95)	0.20
Distance (without interference)	0.04^*^ (1.23)	−0.03^*^ (0.77)	0.03
Distance (with interference)	−0.03 (0.96)	0.00 (1.02)	0.22
**Task 3**			
Accuracy	−0.03 (1.03)	0.06 (0.96)	0.15
Accuracy of telephone number	−0.05 (1.03)	0.03 (0.96)	0.15
Action of prospective memory	−0.04 (1.02)	0.06 (0.97)	0.12
Time	−0.01 (0.97)	−0.06 (1.00)	0.20
Distance	0.02 (0.95)	0.03 (0.95)	0.28
**Task 4**			
Accuracy	−0.05^*^ (1.01)	0.06^*^ (0.99)	0.02
Time	0.04 (1.01)	−0.05 (1.00)	0.11
**Task 5**			
Accuracy	0.05 (0.88)	0.11 (0.70)	0.29
Time	−0.06 (0.95)	−0.08 (0.89)	0.83
Distance	−0.004 (0.98)	−0.09 (0.91)	0.18

*Table reports means and (standard deviations). Subgroup 1: MoCA memory-domain score < median; Subgroup2: MoCA memory-domain score ≥ median*.

* *Denotes significant differences between Subgroup 1 and Subgroup 2*.

#### Task 1—object search

The MoCA memory-domain subgroup 2 outperformed the subgroup 1 in terms of accuracy (*U* = 131977, *p* = 0.004, *d* = 0.21). Subgroup 2 was also faster (*U* = 134535, *p* = 0.03, *d* = 0.13) and covered less distance (*U* = 134141, *p* = 0.025, *d* = 0.13) then the other subgroup while performing this task.

#### Task 2—water the flowers while listening to the radio

The MoCA memory-domain subgroup 1 was similar to the subgroup 2 in terms of accuracy (*U* = 144767, *p* = 0.86), timing (*U* = 139030, *p* = 0.20), and distance with (*U* = 139340, *p* = 0.22) interference. The subgroup 2 covered less distance than the other subgroup in the condition without interference (*U* = 136737, *p* = 0.03, *d* = 0.07).

#### Task 3—make a phone call

The MoCA memory-domain subgroup 1 was similar to the subgroup 2 in terms of accuracy (*U* = 140086, *p* = 0.15), timing (*U* = 139110, *p* = 0.20), distance (*U* = 140440, *p* = 0.28), memory for the phone number (*U* = 140581, *p* = 0.15), and action of prospective memory (*U* = 139708, *p* = 0.12).

#### Task 4—choose the right object

The MoCA memory-domain subgroup 2 outperformed the subgroup 1 in terms of accuracy (*U* = 134722, *p* = 0.021, *d* = 0.11), whereas no differences emerged as regards timing (*U* = 137516, *p* = 0.11).

#### Task 5—find the objects

The MoCA memory-domain subgroup 1 was similar to the subgroup 2 in term of accuracy (*U* = 143884, *p* = 0.29), timing (*U* = 144569, *p* = 0.83), and distance (*U* = 138808, *p* = 0.18).

### Differences in smart aging scores according to MoCA visuospatial-domain subgroups

Means and standard deviations for the five Smart Aging tasks as a function of MoCA visuospatial-domain subgroups are reported in Table 5.

**Table 5:** Z Scores in the five Smart Aging tasks according to MoCA visuospatial-domain subgroups.

	**Subgroup 1** **(*n* = 430)**	**Subgroup 2** **(*n* = 656)**	***p***
**Task 1**			
Accuracy	−0.04 (1.04)	0.10 (0.89)	0.09
Time	0.01 (1.01)	−0.06 (0.96)	0.42
Distance	0.05 (1.02)	−0.01 (0.98)	0.37
**Task 2**			
Accuracy	−0.15^*^ (1.06)	0.06^*^ (0.91)	0.003
Time	0.02 (1.04)	−0.07 (0.94)	0.20
Distance (without interference)	0.001 (0.87)	−0.001 (1.08)	0.59
Distance (with interference)	−0.001 (1.00)	−0.01 (0.98)	0.72
**Task 3**			
Accuracy	−0.12^*^ (1.08)	0.08^*^ (0.94)	0.001
Accuracy of telephone number	−0.11^*^ (1.07)	0.07^*^ (0.93)	0.002
Action of prospective memory	−0.12^*^ (1.06)	0.10^*^ (0.94)	<0.001
Time	0.13^*^ (1.05)	−0.14^*^ (0.94)	<0.001
Distance	0.07 (1.04)	−0.001 (0.88)	0.29
**Task 4**			
Accuracy	−0.21^*^ (1.15)	0.16^*^ (0.85)	<0.001
Time	0.10^*^ (1.06)	−0.09^*^ (0.97)	0.005
**Task 5**			
Accuracy	0.11 (0.63)	0.07 (0.87)	0.60
Time	−0.04 (0.91)	−0.09 (0.92)	0.12
Distance	0.02 (1.02)	−0.10 (0.88)	0.16

*Table reports means and (standard deviations). Subgroup 1: MoCA visuospatial-domain score < median; Subgroup2: MoCA visuospatial-domain score ≥ median*.

* *Denotes significant differences between Subgroup 1 and Subgroup 2*.

#### Task 1—objects search

The MoCA visuospatial-domain subgroup 1 was similar to the subgroup 2 in terms of accuracy (*U* = 132992, *p* = 0.09), timing (*U* = 136944, *p* = 0.42), and distance (*U* = 136495, *p* = 0.37).

#### Task 2—water the flowers while listening to the radio

The MoCA visuospatial-domain subgroup 2 outperformed the subgroup 1 in terms of accuracy (*U* = 126071, *p* = 0.003, *d* = 0.21); whereas the two subgroups were similar for timing (*U* = 134505, *p* = 0.20) and distance without (*U* = 138874, *p* = 0.59) or with (*U* = 139231, *p* = 0.72) interference.

#### Task 3—make a phone call

The MoCA visuospatial-domain subgroup 1 was outperformed by the subgroup 2 in terms of accuracy (*U* = 127914, *p* = 0.001, *d* = 0.20), timing (*U* = 120164, *p* < 0.001, *d* = 0.27), memory for the phone number (*U* = 130024, *p* = 0.002, *d* = 0.18), and action of prospective memory (*U* = 127855, *p* < 0.001, *d* = 0.22), except for distance (*U* = 136033, *p* = 0.29).

#### Task 4—choose the right object

The MoCA visuospatial-domain subgroup 2 outperformed the subgroup 1 in terms of accuracy (*U* = 112464, *p* < 0.001, *d* = 0.37) and timing (*U* = 126730, *p* = 0.005, *d* = 0.19).

#### Task 5—find the objects

The MoCA visuospatial-domain subgroup 1 was similar to the subgroup 2 in terms of accuracy (*U* = 140142, *p* = 0.60), timing (*U* = 133251, *p* = 0.12), and distance (*U* = 133897, *p* = 0.16).

### Differences in smart aging scores according to MoCA executive function (EF) domain subgroups

Means and standard deviations for the five Smart Aging tasks as a function of MoCA EF-domain sugroups are reported in Table 6.

**Table 6:** Z Scores in the five Smart Aging tasks according to MoCA EF-domain subgroups.

	**Subgroup 1** **(*n* = 484)**	**Subgroup 2** **(*n* = 602)**	***p***
**Task 1**			
Accuracy	−0.04^*^ (1.03)	0.12^*^ (0.88)	0.025
Time	0.06^*^ (1.03)	−0.11^*^ (0.94)	0.010
Distance	0.03 (0.99)	0.01 (1.00)	0.65
**Task 2**			
Accuracy	−0.10^*^ (1.02)	0.03^*^ (0.94)	0.007
Time	−0.04 (1.06)	−0.03 (0.91)	0.27
Distance (without interference)	−0.04 (0.95)	0.03 (1.05)	0.52
Distance (with interference)	−0.001 (1.01)	−0.01 (0.98)	0.65
**Task 3**			
Accuracy	−0.07^*^ (1.03)	0.08^*^ (0.95)	0.008
Accuracy of telephone number	−0.09^*^ (1.06)	0.07^*^ (0.94)	0.008
Action of prospective memory	−0.08^*^ (1.04)	0.09^*^ (0.94)	0.005
Time	0.03 (1.05)	−0.08 (0.93)	0.14
Distance	0.04 (0.95)	0.01 (0.95)	0.15
**Task 4**			
Accuracy	−0.14^*^ (0.98)	0.13^*^ (1.00)	<0.001
Time	0.10^*^ (1.02)	−0.10^*^ (0.99)	0.002
**Task 5**			
Accuracy	0.07 (0.90)	0.09 (0.68)	0.93
Time	−0.02 (0.96)	0.11 (0.88)	0.15
Distance	−0.09 (0.93)	−0.02 (0.96)	0.14

*Table reports means and (standard deviations). Subgroup 1: MoCA EF-domain score < median; Subgroup2: MoCA EF-domain score ≥ median*.

* *Denotes significant differences between Subgroup 1 and Subgroup 2*.

#### Task 1—objects search

The MoCA EF-domain subgroup 1 was outperformed by the subgroup 2 in terms of accuracy (*U* = 1235070, *p* = 0.025), *d* = 0.17, and spent less time for completing the task (*U* = 132364, *p* = 0.010, *d* = 0.17). The two subgroups were similar for distances covered (*U* = 143342, *p* = 0.65).

#### Task 2—water the flowers while listening to the radio

The MoCA EF-domain subgroup 1 was outperformed by the subgroup 2 in terms of accuracy (*U* = 131955, *p* = 0.007, *d* = 0.13); whereas the two subgroups were similar for timing (*U* = 140069, *p* = 0.27) and distance without (*U* = 143023, *p* = 0.52) or with (*U* = 143321, *p* = 0.65) interference.

#### Task 3—make a phone call

The MoCA EF-domain subgroup 1 was outperformed by the subgroup 2 in terms of accuracy (*U* = 135410, *p* = 0.008, *d* = 0.15), memory for the phone number (*U* = 136237, *p* = 0.008, *d* = 0.23), and action of prospective memory (*U* = 134821, *p* = 0.005, *d* = 0.17). The two subgroups were similar for timing (*U* = 138144, *p* = 0.14) and distance (*U* = 138825, *p* = 0.15).

#### Task 4—choose the right object

The MoCA EF-domain subgroup 1 was outperformed by the subgroup 2 in terms of accuracy (*U* = 115784, *p* < 0.001, *d* = 0.27) and timing (*U* = 129476, *p* = 0.002, *d* = 0.20).

#### Task 5—find the objects

The MoCA EF-domain subgroup 1 was similar to the subgroup 2 in terms of accuracy (*U* = 145527, *p* = 0.93), timing (*U* = 138352, *p* = 0.15), and distance (*U* = 138152, *p* = 0.14).

### Differences in smart aging scores according to MoCA working memory (WM) domain subgroups

Means and standard deviations for the five Smart Aging tasks as a function of MoCA WM-domain subgroups are reported in Table 7.

**Table 7:** Z Scores in the five Smart Aging tasks according to MoCA WM-domain subgroups.

	**Subgroup 1** **(*n* = 56)**	**Subgroup 2** **(*n* = 1,030)**	***p***
**Task 1**			
Accuracy	−0.05 (1.08)	0.05 (0.95)	0.57
Time	0.25^*^ (0.86)	−0.05^*^ (0.99)	0.02
Distance	0.19 (1.02)	0.01 (0.99)	0.32
**Task 2**			
Accuracy	−0.03 (1.20)	−0.02 (0.97)	0.59
Time	0.27^*^ (1.04)	−0.05^*^ (0.98)	0.014
Distance (without interference)	−0.14 (0.49)	0.01 (1.02)	0.64
Distance (with interference)	0.21 (1.44)	−0.02 (0.96)	0.90
**Task 3**			
Accuracy	−0.18 (1.22)	0.03 (0.98)	0.25
Accuracy of telephone number	−0.21 (1.13)	0.01 (0.99)	0.11
Action of prospective memory	−0.12 (1.07)	0.02 (0.99)	0.30
Time	0.13 (1.02)	−0.04 (0.99)	0.23
Distance	−0.10 (0.80)	0.03 (0.95)	0.53
**Task 4**			
Accuracy	−0.10 (1.00)	0.02 (0.10)	0.34
Time	0.20 (0.94)	−0.02 (1.01)	0.11
**Task 5**			
Accuracy	−0.17^*^ (1.49)	0.10^*^ (0.73)	0.042
Time	0.26^*^ (1.04)	−0.09^*^ (0.90)	0.003
Distance	0.08 (0.95)	−0.06 (0.94)	0.18

*Table reports means and (standard deviations). Subgroup 1: MoCA WM-domain score < median; Subgroup2: MoCA WM-domain score ≥ median*.

* *Denotes significant differences between Subgroup 1 and Subgroup 2*.

#### Task 1—objects search

The MoCA WM-domain subgroup 2 used less time for completing Task 1 than the subgroup 1 (*U* = 23683, *p* = 0.02, *d* = 0.81). The two subgroups were similar for accuracy (*U* = 27645, *p* = 0.57) and distance covered (*U* = 26549, *p* = 0.32).

#### Task 2—water the flowers while listening to the radio

The MoCA WM-domain subgroup 1 was slower than subgroup (*U* = 23229, *p* = 0.014, *d* = 0.76); whereas the two subgroups were similar for accuracy (*U* = 27605, *p* = 0.59) and distance without (*U* = 27983, *p* = 0.64) or with (*U* = 26548, *p* = 0.90) interference.

#### Task 3—make a phone call

The MoCA WM-domain subgroup 1 was similar to the subgroup 2 for all indices: accuracy (*U* = 26882, *p* = 0.25), memory for the phone number (*U* = 26284, *p* = 0.11), action of prospective memory (*U* = 27085, *p* = 0.30), timing (*U* = 26100, *p* = 0.23), and distance (*U* = 27492, *p* = 0.53).

#### Task 4—choose the right object

The MoCA WM-domain subgroup 1 did not differ from the subgroup 2 in terms of accuracy (*U* = 26812, *p* = 0.34) and timing (*U* = 25143, *p* = 0.11).

#### Task 5—find the objects

The MoCA WM-domain subgroup 1 was less accurate (*U* = 27285, *p* = 0.042, *d* = 0.23) and slower (*U* = 22046, *p* = 0.003, *d* = 0.36) than the subgroup 2; whereas the two subgroups were similar in terms of distance (*U* = 25801, *p* = 0.18) covered while performing this task.

### Differences in smart aging scores according to MoCA language-domain subgroups

Means and standard deviations for the five Smart Aging tasks as a function of MoCA language-domain subgroups are reported in Table 8.

**Table 8:** Z Scores in the five Smart Aging tasks according to MoCA language-domain subgroups.

	**Subgroup 1** **(*n* = 119)**	**Subgroup 2** **(*n* = 967)**	***p***
**Task 1**			
Accuracy	0.05 (0.98)	0.05 (0.95)	0.82
Time	−0.02 (0.91)	−0.04 (0.99)	0.92
Distance	−0.04 (0.97)	0.02 (1.00)	0.52
**Task 2**			
Accuracy	−0.08 (1.14)	−0.01 (0.96)	0.84
Time	0.10 (1.03)	−0.05 (0.97)	0.14
Distance (without interference)	0.05 (0.88)	−0.01 (1.02)	0.50
Distance (with interference)	−0.01 (1.16)	−0.01 (0.97)	0.99
**Task 3**			
Accuracy	−0.12 (1.02)	0.03 (0.99)	0.07
Accuracy of telephone number	−0.06 (1.05)	0.01 (0.99)	0.47
Action of prospective memory	−0.12 (1.06)	0.03 (0.98)	0.13
Time	0.12 (1.04)	−0.05 (0.98)	0.06
Distance	0.11 (0.93)	0.01 (0.95)	0.05
**Task 4**			
Accuracy	0.02 (0.98)	0.01 (1.00)	0.89
Time	0.12 (1.03)	−0.03 (1.00)	0.13
**Task 5**			
Accuracy	0.07 (0.93)	0.09 (0.77)	0.85
Time	0.05 (0.93)	−0.08 (0.91)	0.032
Distance	−0.04 (0.96)	−0.05 (0.94)	0.97

Table reports means and (standard deviations). Subgroup 1: MoCA language-domain score < median; Subgroup2: MoCA language-domain score ≥ median. ^*^ Denotes significant differences between Subgroup 1 and Subgroup 2.

#### Task 1—objects search

The MoCA language-domain subgroup 1 was similar to the subgroup 2 in all indices: accuracy (*U* = 56867, *p* = 0.82), timing (*U* = 57199, *p* = 0.92), and distances covered (*U* = 55455, *p* = 0.52).

#### Task 2—water the flowers while listening to the radio

The MoCA language-domain subgroup 1 did not differ from the subgroup 2 in terms of accuracy (*U* = 56908, *p* = 0.84), timing (*U* = 52769, *p* = 0.14), and distance without (*U* = 55787, *p* = 0.50) or with (*U* = 57503, *p* = 0.99) interference.

#### Task 3—make a phone call

The MoCA language-domain subgroup 1 was similar to the subgroup 2 for all indices: accuracy (*U* = 53096, *p* = 0.07), memory for the phone number (*U* = 55906, *p* = 0.47), action of prospective memory (*U* = 53875, *p* = 0.13), timing (*U* = 51524, *p* = 0.063), and distance (*U* = 51622, *p* = 0.051).

#### Task 4—choose the right object

The MoCA language-domain subgroup 1 did not differ from the subgroup 2 in terms of accuracy (*U* = 57105, *p* = 0.89) and timing (*U* = 52578, *p* = 0.13).

#### Task 5—find the object

The MoCA language-domain subgroup 1 was slower (*U* = 50598, *p* = 0.032) while performing Task 5 than the subgroup 2; whereas the two subgroups were similar in term of accuracy (*U* = 57329, *p* = 0.85) and distance (*U* = 57393, *p* = 0.97) covered while performing this task.

### Differences in smart aging scores according to MoCA orientation-domain subgroups

Means and standard deviations for the five Smart Aging tasks as a function of MoCA orientation-domain subgroups are reported in Table 9.

**Table 9:** Z Scores in the five Smart Aging tasks according to MoCA orientation-domain subgroups.

	**Subgroup 1** **(*n* = 78)**	**Subgroup 2** **(*n* = 1,008)**	***p***
**Task 1**			
Accuracy	0.03 (0.88)	0.05 (0.96)	0.65
Time	0.08 (1.03)	−0.04 (0.98)	0.35
Distance	0.05 (1.04)	0.01 (0.99)	0.70
**Task 2**			
Accuracy	−0.05 (1.04)	−0.02 (0.98)	0.96
Time	0.14 (1.04)	−0.05 (0.98)	0.10
Distance (without interference)	0.01 (0.99)	−0.01 (1.00)	0.75
Distance (with interference)	0.08 (1.02)	−0.01 (0.99)	0.74
**Task 3**			
Accuracy	−0.02 (1.00)	0.02 (0.99)	0.66
Accuracy of telephone number	−0.13 (1.09)	0.01 (0.99)	0.21
Action of prospectivememory	−0.01 (1.01)	0.02 (0.99)	0.82
Time	0.04 (1.17)	−0.04 (0.98)	0.89
Distance	0.03 (0.45)	0.02 (0.98)	0.26
**Task 4**			
Accuracy	−0.22^*^ (1.01)	0.03^*^ (0.99)	0.008
Time	−0.07 (0.98)	−0.01 (1.01)	0.49
**Task 5**			
Accuracy	0.10 (0.53)	0.09 (0.80)	0.57
Time	0.01 (0.96)	−0.08 (0.91)	0.32
Distance	−0.06 (0.95)	−0.05 (0.94)	0.75

*Table reports means and (standard deviations). Subgroup 1: MoCA orientation-domain score < median; Subgroup2: MoCA orientation-domain score ≥ median*.

* *Denotes significant differences between Subgroup 1 and Subgroup 2*.

#### Task 1—object search

The MoCA orientation-domain subgroup 1 was similar to the subgroup 2 in all indices: accuracy (*U* = 38190, *p* = 0.65), timing (*U* = 36827, *p* = 0.35), and distances covered (*U* = 38291, *p* = 0.70).

#### Task 2—water the flowers while listening to the radio

The MoCA orientation-domain subgroup 1 did not differ from the subgroup 2 in terms of accuracy (*U* = 39182, *p* = 0.96), timing (*U* = 34975, *p* = 0.10), and distance without (*U* = 38635, *p* = 0.75) or with (*U* = 38414, *p* = 0.74) interference.

#### Task 3

The MoCA orientation-domain subgroup 1 was similar to the subgroup 2 for all indices: accuracy (*U* = 38444, *p* = 0.66), memory for the phone number (*U* = 36993, *p* = 0.21), action of prospective memory (*U* = 38865, *p* = 0.82), timing (*U* = 38962, *p* = 0.89), and distance (*U* = 36510, *p* = 0.26).

#### Task 4—choose the right object

The MoCA orientation-domain subgroup 1 was outperformed by the subgroup 2 in terms of accuracy (*U* = 32751, *p* = 0.008, *d* = 0.25); whereas the two subgroups were similar for timing (*U* = 37453, *p* = 0.49).

#### Task 5—find the object

The MoCA orientation-domain subgroup 1 did not differ from subgroup2 for accuracy (*U* = 38806, *p* = 0.57), timing (*U* = 36635, *p* = 0.32), and distance (*U* = 38447, *p* = 0.75) covered while performing this task.

## Discussion

The main goal of the present study was to validate the Smart Aging platform by examining its performance in assessing global cognitive functions and specific cognitive domains in a sample of more than 1,000 adults aged over 50 years. To this end, we first assessed performances in several Smart Aging indices according to the MoCA global performance. Second, we evaluated whether performances in the several game tasks changed according to the functioning in the several cognitive domains making up the MoCA.

For what concerns global performance, most of the tasks and indices of the game were sensitive to differences according to the global cognitive functions. Among all indices, those more impacted by differences were accuracy and time for completing each task. What is particularly interesting here is the fact that the great majority of these differences involved not only extreme groups—i.e., those scoring below 27 *or* above 29 on the MoCA—but also older adults scoring *between* these two intervals. Hence, this finding suggests that the assessment made with Smart Aging was in line with that made using classical assessment instruments. In particular, among all tasks, we found that accuracy and time in Task 2 and in Task 4 seem to be those indices more associated to the MoCA performance. Such a finding seems to suggest that these two tasks could be considered a good proxy for global cognitive mechanisms. However, we found less differences across MoCA groups for what concerns the total amount of distance covered while performing the task. A reason for this finding could be that the virtual scenario was *per se* too small for detecting differences in terms of distance. When measuring ROC curve and AUC on total scores of the five tasks as well as on the Smart Aging total score, we found a poor discriminative capacity of this platform. This finding is, however, not surprising given that our data have been collected on a sample of healthy participants. The future inclusion of cognitive-impaired participants will hopefully demonstrate the “true” discriminative power of this game.

Our results are also interesting as they show what specific cognitive functions were contributing to the achievement of each Smart Aging task. Task 1, Task 4, and Task 5 were originally conceived as measures of memory; whereas Task 2 and Task 3 as measures of executive processes working memory and visual spatial abilities. When considering single MoCA cognitive domains, we found in part a correspondence with our expectations. In particular, Task 1 and Task 4 resulted particularly associated to memory, whereas Task 2 and Task 3 to executive functions, working memory, and visual-spatial abilities. By contrast, language and orientation were practically not associated to any task, which is not surprising. In the case of language, it was not expected to be involved in any task. As regards orientation, we expected that this ability could be necessary for moving in the virtual scenario but probably such a finding could be related to the same lack of differences across older adults we found for what concerns distance covered while performing tasks, as explained above. A further explanation could take into account the disparity of proportion of older adults failing in these two cognitive domains with respect to the other domains of the MoCA. However, the correspondence resulted for cognitive domains of memory, executive mechanisms, and visual-spatial processes with the classical neuropsychological assessments suggests that Smart Aging could be easily administered for evaluate these domains, being those abilities mostly supporting instrumental activities of daily living (Schmitter-Edgecombe et al., 2009).

In line with this, we also found that several tasks were associated to different cognitive domains, which can be explained by the multitasking features that are also requested to perform everyday life activities (Fortin et al., 2003). In particular, Task 1, 2, and 3 are those more associated to several specific cognitive domains, such as memory, visuo-spatial abilities, executive functions, and working memory, as assessed by the MoCA cognitive subdomains. This finding is particularly interesting as it highlights the importance of assessing multiple cognitive abilities together at the same time as in real life settings instead of using single tests that do not allow the detection of their synergistic functioning. Classic neuropsychological evaluations are instead characterized by the assessment of individual cognitive abilities that could be differently affected by aging and also act differently when working together in daily life activities (Logan and Barber, 2013). This same finding is confirmed by the different pattern or results obtained when grouping our population by considering the MoCA as an index of global or specific cognitive functions. Hence, we believe that the serious games devised as assessment tools in Smart Aging platform have the strength to allow to evaluate how cognitive functions act together as a whole in a more ecological manner. Hence, they could be considered optimal predictors of daily life functioning. This issue is particularly critical in order to have a real view of older adults functioning outside of the laboratory setting. Future studies will extend these findings in clinical populations by comparing scores in Smart Aging with those of instruments assessing daily life functioning.

To date, we validated this platform on a healthy population of older adults. Future studies are planned and necessary to evaluate the performance of the Smart Aging platform in the screening of pre-dementia and dementia conditions. An additional development should be to set tasks in order to be easily playable for independent use, so that specific cognitive abilities should be evaluated according to the diagnostic question. Finally, just developing other scenarios and tasks with different levels of complexity, this platform could be easily used for monitoring older adults' functioning at distance and also as a rehabilitative tool. Our hope, in the future, is that this platform could be easily administered as a substitute of the classical functional, behavioral, and neuropsychological assessments, instead of a complement of them.

In conclusion, the present findings suggest that the Smart Aging SG platform could constitute a powerful screening tool for cognitive functions on a wide scale. Virtual reality and interactive video gaming represent indeed new promising ways for assessing cognitive mechanisms (Christiansen et al., 1998; Rizzo et al., 1998; Davies et al., 1999; Riva et al., 1999; Rose et al., 1999; Jack et al., 2001; Zhang et al., 2001; Kang et al., 2008). These tools involve computer-based programs being designed to simulate real life situations, and having important advantages over traditional approaches: they are more friendly, ecological and motivating for the end-users, and their less time- and resource-consuming for the professional figures. In this field, the challenge is to develop a tool that, while exploiting the latest technological solutions, may also be used by users with minimal computer experience, as older adults usually are. Previous studies have indeed reported that computerized vs. examiner-administered testing may be different in computer-competent vs. computer-naïve populations (Feldstein et al., 1999) as a result of significant individual differences in computer use and familiarity (Iverson et al., 2009). To cope with this issue, in the Smart Aging platform the movements in the environment were performed using a touch screen: this choice is supported by the data gathered in previous testing (Zucchella et al., 2014) and by literature data showing that the touch screen has proven to be easier to learn and more intuitive than a mouse for users with minimal computer experience or some level of cognitive impairment (Cernich et al., 2007). We also choose a non-immersive interaction that could help older adults to become comfortable with the virtual environment and to avoid adverse risks, such as nausea and headache, related to immersive interactions (Flynn et al., 2003). In clinical practice, indeed, it is very important to avoid that individuals with less computer familiarity are misdiagnosed as having frank cognitive impairment. The fact that our older adults' performances were associated to their actual cognitive state seems to suggest the diagnostic accuracy of this computerized neurocognitive testing. Furthermore, unlike others tools (e.g., Oliveira et al., 2016) that investigate a specific cognitive function, a strength of Smart Aging is that it allows the evaluation of multiple cognitive domains (memory, executive functions, etc.) that could characterize the different patterns of cognitive decline. In addition, we tested the association between serious games performances and cognitive abilities on a very numerous sample of over 50 adults. Many studies indeed in the existing literature had smaller sample size and should be considered only preliminary with respect to the validation of these platforms (e.g., McGee et al., 2000; Boletsis and McCallum, 2016; Oliveira et al., 2016; Vallejo et al., 2017).

Smart Aging is a SG platform realized through a strong collaboration between game developers and neuropsychologist experts for assessing cognitive functions. The results of this study suggest that this platform can be reliably used in healthy middle-aged/older adults and represent an innovative tool for clinicians for evaluating cognitive mechanisms thanks to the many advantages that virtual realities offer in comparison to the traditional screening tests.

## Ethics statement

This study was carried out in accordance with the recommendations of the Ethical Committee of the San Matteo Hospital in Pavia with written informed consent from all subjects. All subjects gave written informed consent in accordance with the Declaration of Helsinki. The protocol was approved by the Ethical Committee of the San Matteo Hospital.

## Author contributions

SBo designed the game, carried out the statistical analyses, and wrote the manuscript. CT assisted research design, statistical analyses, interpretation of the results, and manuscript writing. ML collected the data. CZ and EC designed the game, assisted with the selection of clinical assessments used in the study, and interpreted the data. SBe and ES assisted with the selection of clinical assessments. SP and PC supervised data collection and storage, and assisted in data analyses. DT developed the game. TV and GS supervised the entire study. All authors did read and approve the final version of the manuscript.

### Conflict of interest statement

The authors declare that the research was conducted in the absence of any commercial or financial relationships that could be construed as a potential conflict of interest.

## References

[B1] Aalbers T. Baars M. A. Olde Rikkert M. G. Kessels R. P. (2013). Puzzling with online games (BAM-COG): reliability, validity, and feasibility of an online self-monitor for cognitive performance in aging adults. J. Med. Internet Res. 15:e270. 10.2196/jmir.2860 24300212 PMC3868977

[B2] Anguera J. A. Boccanfuso J. Rintoul J. L. Al-Hashimi O. Faraji F. Janowich J.. (2013). Video game training enhances cognitive control in older adults. Nature 501, 97–101. 10.1038/nature12486 24005416 PMC3983066

[B3] Boletsis C. McCallum S. (2016). Smartkuber: a serious game for cognitive health screening of elderly players. Games Health J 5, 241–251. 10.1089/g4h.2015.0107 27192473

[B4] Cernich A. N. Brennana D. M. Barker L. M. Bleiberg J. (2007). Sources of error in computerized neuropsychological assessment. Arch. Clin. Neuropsychol. 22, 39–48. 10.1016/j.acn.2006.10.004 17097851

[B5] Charsky D. (2010). From edutainment to serious games: a change in the use of game characteristics. Sage J. 5, 177–198. 10.1177/1555412009354727

[B6] Chaytor N. Schmitter-Edgecombe M. (2003). The ecological validity of neuropsychological tests: a review of the literature on everyday cognitive skills. Neuropsychol. Rev. 13, 181–97. 10.1023/B:NERV.0000009483.91468.fb 15000225

[B7] Christiansen C. Abreu B. Ottenbacher K. Huffman K. Masel B. Culpepper R. (1998). Task performance in virtual environments used for cognitive rehabilitation after traumatic brain injury. Arch. Phys. Med. Rehabil. 79, 888–892. 10.1016/S0003-9993(98)90083-1 9710158

[B8] Ciesielska N. Sokolowski R. Mazur E. Podhorecka M. Polak-Szabela A. Kedziora-Kornatowska K. (2016). Is the Montreal Cognitive Assessment (MoCA) test better suited than the Mini-Mental State Examination (MMSE) in mild cognitive impairment (MCI) detection among people aged over 60? Meta-analysis. Psychiatr. Pol. 50, 1039–1052. 10.12740/PP/45368 27992895

[B9] Davies R. C. Hohansson G. Boschian K. Lindén A. Minör U. Sonesson B. (1999). A practical example using VR in the assessment of brain injury. Int. J. Virtual Real. 4, 61–68.

[B10] Davison S. M. C. Deeprose C. Terbeck S. (2017). A comparison of immersive virtual reality with traditional neuropsychological measures in the assessment of executive functions. Acta Neuropsychol. 9, 1–11. 10.1017/neu.2017.14 28482936

[B11] Faul F. Erdfelder E. Lang A.-G. Buchner A. (2007). G^*^Power 3: a flexible stati-stical power analysis program for the social, behavioral, and biomedical sciences. Behav. Res. Methods 39, 175–191. 10.3758/BF03193146 17695343

[B12] Feldstein S. N. Keller F. R. Portman R. E. Durham R. L. Klebe K. J. Davis H. P. (1999). A comparison of computerized and standard versions of the Wisconsin Card Sorting Test. Clin. Neuropsychol. 13, 303–313. 10.1076/clin.13.3.303.1744 10726602

[B13] Fernandez-Montenegro J. M. Argyriou V. (2017). Cognitive evaluation for the diagnosis of Alzheimer's disease based on turing test and virtual environments. Physiol. Behav. 173, 42–51. 10.1016/j.physbeh.2017.01.034 28137425

[B14] Flynn D. Van Schaik P. Blackman T. Femcott C. Hobbs B. Calderon C. (2003). Developing a virtual reality-based methodology for people with dementia: a feasibility study. Cyberpsychol. Behav. 6, 591–611. 10.1089/109493103322725379 14756925

[B15] Fortin S. Godbout L. Braun C. M. (2003). Cognitive structure of executive deficits in frontally lesioned head trauma patients performing activities of daily living. Cortex 39, 273–291. 10.1016/S0010-9452(08)70109-6 12784889

[B16] Gamberini L. Alcaniz M. Barres G. Fabregat M. Ibanez F. Prontu L. (2006). Cognition, technology and games for the elderly: an introduction to ELDERGAMES Project. Psychnol. J. 4, 285–308.

[B17] Gluhm S. Goldstein J. Loc K. Colt A. Van Liew C. Corey-Bloom J. (2013). Cognitive performance on the mini-mental state examination and the montreal cognitive assessment across the healthy adult lifespan. Cogn. Behav. Neurol. 26, 1–5. 10.1097/WNN.0b013e31828b7d26 23538566 PMC3638088

[B18] Ismail Z. Rajji T. K. Shulman K. I. (2010). Brief cognitive screening instruments: an update. Int. J. Geriatr. Psychiatry 25, 111–120. 10.1002/gps.2306 19582756

[B19] Iverson G. L. Brooks B. L. Ashton V. L. Johnson L. G. Gualtieri C. T. (2009). Does familiarity with computers affect computerized neuropsychological test performance? J. Clin. Exp. Neuropsychol. 31, 594–604. 10.1080/13803390802372125 18972312

[B20] Jack D. Boian R. Merians A. S. Tremaine M. Burdea G. C. Adamovich S. V.. (2001). Virtual reality-enhanced stroke rehabilitation. IEEE Trans. Neural Syst. Rehabil. Eng. 9, 3–318. 10.1109/7333.948460 11561668

[B21] Kang Y. J. Ku J. Han K. Kim S. I. Yu T. W. Lee J. H.. (2008). Development and clinical trial of virtual reality-based cognitive assessment in people with stroke: preliminary study. Cyberpsychol. Behav. 11, 329–339. 10.1089/cpb.2007.0116 18537503

[B22] Logan G. D. Barber CY. (2013). On the ability to inhibit complex thoughts: a stop-signal study of arithmetic. Bull. Psychon. Soc. 23, 371–373. 10.3758/BF03330187

[B23] Manera V. Petit P. D. Derreumaux A. Orvieto I. Romagnoli M. Lyttle G.. (2015). ‘Kitchen and cooking,’ a serious game for mild cognitive impairment and Alzheimer's disease: a pilot study. Front. Aging Neurosci. 7:24. 10.3389/fnagi.2015.00024 25852542 PMC4362400

[B24] McGee J. S. Van Der Zaag C. Buckwalter J. G. Thiebaux M. Van Rooyen A. Neumann U. (2000). Issues for the assessment of visuospatial skills in older adults using virtual environment technology. Cyberpsychol. Behav. 3, 469–482. 10.1089/10949310050078931

[B25] Nasreddine Z. S. Phillips N. A. Bédirian V. Charbonneau S. Whitehead V. Collin I.. (2005). The Montreal Cognitive Assessment, MoCA: a brief screening tool for mild cognitive impairment. J. Am. Geriatr. Soc. 53, 695–699. 10.1111/j.1532-5415.2005.53221.x 15817019

[B26] Oliveira C. R. Lopes Filho B. J. Sugarman M. A. Esteves C. S. Lima M. M. Moret-Tatay C.. (2016). Development and feasibility of a virtual reality task for the cognitive assessment of older adults: the ECO-VR. Span. J. Psychol. 19:e95. 10.1017/sjp.2016.96 27955716

[B27] Pazzi S. Falleri V. Puricelli S. von Barnekov A. Tost D. Grau S. (2014). A Serious Games platform for early diagnosis of mild cognitive impairments, in Games for Health, eds Schouten B. Fedtke S. Schijven M. Vosmeer M. Gekker A. (Wiesbaden: Springer Fachmedien), 110–113.

[B28] Riva G. Rizzo A. Alpini D. Attree E. A. Barbieri E. Bertella L.. (1999). Virtual environments in the diagnosis, prevention and intervention of age related diseases. Cyberpsychol. Behav. 2, 577–592. 10.1089/cpb.1999.2.577 19178205

[B29] Rizzo A. A. Buckwalter G. Neumann U. Kesselman C. Thiebaux M. (1998). Basic issues in the application of virtual reality for the assessment and rehabilitation of cognitive impairments and functional disability. Cyberpsychol. Behav. 1, 59–78. 10.1089/cpb.1998.1.59

[B30] Robert P. H. König A. Amieva H. Andrieu S. Bremond F. Bullock R.. (2014). Recommendations for the use of serious games in people with Alzheimer's disease, related disorders and frailty. Front. Aging Neurosci. 6:54. 10.3389/fnagi.2014.00054 24715864 PMC3970032

[B31] Rose F. D. Brooks B. M. Attree E. A. Parslow D. M. Leadbetter A. G. McNeil J. E.. (1999). A preliminary investigation into the use of virtual environments in memory retraining after vascular brain injury: indications for future strategy? Disabil. Rehabil. 21, 548–554. 10.1080/096382899297206 10608651

[B32] Schmitter-Edgecombe M. Woo E. Greeley D. R. (2009). Characterizing multiple memory deficits and their relation to everyday functioning in individuals with mild cognitive impairment. Neuropsychology 23, 168–177. 10.1037/a0014186 19254090

[B33] Stopford C. L. Thompson J. C. Neary D. Richardson A. M. Snowden J. S. (2012). Working memory, attention, and executive function in Alzheimer's disease and frontotemporal dementia. Cortex 48, 429–446. 10.1016/j.cortex.2010.12.002 21237452

[B34] Tarnanas I. Papagiannopoulos S. Kazis D. Wiederhold M. Wiederhold B. Tsolaki M. (2015). Reliability of a novel serious game using dual-task gait profiles to early characterize aMCI. Front. Aging Neurosci. 7:50. 10.3389/fnagi.2015.00050 25954193 PMC4406069

[B35] Tarnanas I. Schlee W. Tsolaki M. Müri R. Mosimann U. Nef T. (2013). Ecological validity of virtual reality daily living activities screening for early dementia: longitudinal study. JMIR Serious Games 1:e1. 10.2196/games.2778 25658491 PMC4307822

[B36] Tarnanas I. Tsolaki M. Nef T. Müri R. Mosimann U. (2014). Can a novel computerized cognitive screening test provide additional information for early detection of Alzheimer's disease? Alzheimers Dement. 10, 790–798. 10.1016/j.jalz.2014.01.002 24656838

[B37] Tong T. Chignell M. Tierney M. C. Lee J. (2016). A serious game for clinical assessment of cognitive status: validation study. JMIR Serious Games 27:e7 10.2196/games.5006 PMC4902858 27234145

[B38] Tost D. Pazzi S. von Barnekow A. Felix E. Puricelli S. Bottiroli S. (2014). SmartAgeing: a 3D serious game for early detection of mild cognitive impairments, in Proceedings of the 8th International Conference on Pervasive Computing Technologies for Healthcare (PervasiveHealth ′14). ICST, Brussels, 294–297.

[B39] Tost D. von Barnekow A. Felix E. Pazzi S. Puricelli S. Bottiroli S. (2015). Early detection of cognitive impairments with the Smart Ageing serious game, ICTs for Improving Patients Rehabilitation Research Techniques, Vol. 515, eds Fardoun H. Penichet V. R. Alghazzawi D. (Berlin, Heidelberg: Springer), 183–195. 10.1007/978-3-662-48645-0_16

[B40] Valladores-Rodríguez S. Pérez-Rodríguez R. Anido-Rifón L. Fernández-Iglesias M. (2016). Trends on the application of serious games to neuropsychological evaluation: a scoping review. J. Biomed. Informatics 64, 296–319. 10.1016/j.jbi.2016.10.019 27815228

[B41] Vallejo V. Wyss P. Cheshama A. Mitache A. V. Müri R. M. Mosimann U. O. (2017). Evaluation of a new serious game based multitasking assessment tool for cognition and activities of daily living: Comparison with a real cooking task. Comp. Hum. Behav. 70, 500–506. 10.1016/j.chb.2017.01.021

[B42] Wiemeyer J. Kliem A. (2012). Serious games in prevention and rehabilitation—a new panacea for elderly people? Eur. Rev. Aging Phys. Act. 9, 41–50. 10.1007/s11556-011-0093-x

[B43] Wild K. Howiesona D. Webbeb F. Seelyea A. Kaye J. (2008). Status of computerized cognitive testing in aging: a systematic review. Alzheimers Dement. 4, 428–437. 10.1016/j.jalz.2008.07.003 19012868 PMC2645803

[B44] Zhang L. Abreu B. Masel B. Scheibel R. S. Christiansen C. H. Huddleston N.. (2001). Virtual reality in the assessment of selected cognitive function after brain injury. Am. J. Phys. Med. Rehabil. 80, 597–604. 10.1097/00002060-200108000-00010 11475481

[B45] Zucchella C. Sinforiani E. Tassorelli C. Cavallini E. Tost-Pardell D. Grau S.. (2014). Serious games for screening pre-dementia conditions: from virtuality to reality? A pilot project. Funct. Neurol. 29, 153–158. 10.11138/FNeur/2014.29.3.153 25473734 PMC4264781

[B46] Zygouris S. Tsolaki M. (2014). Computerized cognitive testing for older adults: a review. Am. J. Alzheimers. Dis. Other Demen. 30, 13–28. 10.1177/1533317514522852 24526761 PMC10852880

[B47] Zygouris S. Giakoumis D. Votis K. Doumpoulakis S. Ntovas K. Segkouli S.. (2015). Can a virtual reality cognitive training application fulfill a dual role? using the virtual supermarket cognitive training application as a screening tool for mild cognitive impairment. J. Alzheimers Dis. 44, 1333–1347. 10.3233/JAD-141260 25428251

